# Patients With a History of Spine Surgery Receiving Chiropractic Spinal Manipulation in US Academic Health Centers: A Cross-Sectional Cohort Study

**DOI:** 10.7759/cureus.37216

**Published:** 2023-04-06

**Authors:** Robert J Trager, Pratheek S Makineni, Timothy J Williamson

**Affiliations:** 1 Chiropractic, Connor Whole Health, University Hospitals Cleveland Medical Center, Cleveland, USA; 2 Medicine, Case Western Reserve University, Cleveland, USA; 3 Chiropractic, UCHealth Integrative Medicine Center, University of Colorado Hospital, Denver, USA

**Keywords:** spinal fusion, low back pain, neck pain, surgical procedure, failed back surgery syndrome, chiropractic, spinal manipulation

## Abstract

Introduction: The number and characteristics of patients with previous spine surgery receiving chiropractic spinal manipulation (CSM) are largely unknown. This study aimed to explore the proportion of patients receiving CSM with a history of spine surgery, the characteristics of these patients, and the treatments received compared to a broader population of patients receiving CSM.

Methods: We queried a 110-million-patient United States (US) network of aggregated records and claims data from patients attending integrated academic health centers (TriNetX, Inc.) on March 6, 2023, yielding data spanning 2013-2023. We identified two patient groups: (1) those receiving CSM and (2) a subset receiving CSM with prior spine surgery. We compared baseline characteristics and treatments received over a one-year follow-up after CSM.

Results: Of the 81,291 patients receiving CSM, 8,808 (10.8%) had at least one prior spine surgery. Patients with prior spine surgery receiving CSM were older, more often female, more often non-Hispanic/Latino and White, less often Black, had a greater body mass index, and had a higher prevalence of low back and neck pain compared to the broader CSM cohort (*p*<0.0001 for each). Those with prior spine surgery were also more likely to receive multiple medications, physiotherapy procedures, and spinal injections (*p*<0.0001 for each).

Conclusion: Patients receiving CSM with a history of spine surgery comprise a substantial proportion of CSM patients in large US academic health centers. This subset of patients differs characteristically from the broader CSM population and more often receives medications, physiotherapy, and spinal injections. Further research is needed to examine the safety and efficacy of CSM in this population, given the high proportion of patients and limited research on this topic.

## Introduction

Chiropractors are healthcare providers who commonly treat spinal disorders such as neck and low back pain. Evidence has emerged that chiropractors also care for patients with persistent or recurrent pain after spine surgery using manual (i.e., hands-on) therapies, including spinal manipulation [1-3]. However, there needs to be more understanding of the proportion and characteristics of patients with a history of spine surgery who seek care from chiropractors for neck and back pain.

Chiropractic spinal manipulation (CSM) includes a variety of thrust (i.e., high-velocity, low-amplitude impulse) and non-thrust (i.e., mobilization) techniques directed to the joints of the spine [4]. While evidence has supported the safety and efficacy of CSM for low back pain and neck pain [5-7], there is only limited evidence of its utility for patients with previous spine surgery, mainly in retrospective observational studies [1,2,8]. One reason is that randomized controlled trials examining CSM often exclude patients with previous spine surgery [4]. Given that these patients may have chronic postsurgical symptoms and potential contraindications to CSM (e.g., instability or internal fixation/stabilization devices with adjacent segment disease) [4], it is not clear if the benefits of CSM translate to this population. Accordingly, further research is needed to quantify and characterize this population to facilitate the design of prospective trials.

Few studies have reported the proportion of patients with previous spine surgery receiving chiropractic care. In a recent retrospective chart review of 20 chiropractic clinics published in 2022, patients with prior lumbar spine surgery made up 0.5% (i.e., 31/6,589) of patients with low back pain during three years [2]. In another retrospective study, patients with prior lumbar spine surgery accounted for 0.2% (i.e., 7/3,531) of new patients presenting to a chiropractic teaching clinic over four years [9]. Finally, in a chart review including 759 patients with chronic low back pain, only one patient had a previous laminectomy (i.e., 0.1%) [10].

In addition, few studies have described the characteristics of patients with spine surgery receiving CSM. Across the few observational studies reporting such information, patients with previous lumbar spine surgery receiving CSM are typically at least 50 years old without any clear sex predominance [2,11,12]. Even less is known regarding the characteristics of those with previous cervical spine surgery receiving CSM. A recent systematic review of manual therapy use following cervical spine surgery only identified three case reports, each describing a single patient receiving spinal manipulation [8].

Some research has suggested that patients with previous spine surgery receive CSM as part of a broader multimodal treatment plan, including exercises (e.g., strengthening, stretching) and other passive physiotherapy modalities [1,2,13,14]. In a retrospective study in which patients received CSM for persistent low back pain after lumbar spine surgery (n=31), 65% also received ultrasound thermotherapy, electrotherapy, and cryotherapy [2]. Such patients may aim to exhaust several conservative treatment options to manage their spinal pain and avoid re-operation [13,14]. As the application of CSM alongside other therapies needs to be better described, the current study, which incorporates data from integrated academic health centers, should provide a better description of such data.

As described, there needs to be more understanding of the relative number and features of patients with previous spinal surgery receiving CSM. Thus, this study will further describe these patients using a large United States (US) health records database. Our objectives are to explore the proportion of patients receiving CSM that have had spine surgery. Of these patients, their characteristics (e.g., demographics, anthropometrics, symptom prevalence) and other treatments received compared to the broader population of patients receiving CSM.

## Materials and methods

This study implemented a cross-sectional cohort design using real-world data from the research network TriNetX (Cambridge, MA, USA). This network includes de-identified, aggregated, linked medical records and claims data from over 110 million patients and 75 healthcare organizations, typically large, academically affiliated health centers with associated specialty care and outpatient facilities. To protect patient privacy, specific participating organizations remain anonymous. This study was deemed Not Human Subjects Research by the University Hospitals Institutional Review Board (Cleveland, OH, USA, STUDY20230066).

Patient data from ten years to one year before the query date (i.e., data range of 2013-2022; query date of March 6, 2023) were queried to allow for one year of follow-up data. Patients of any age were included at their first receipt of CSM and were categorized into two groups (1) those receiving CSM and (2) a subset of those with previous spine surgery. Spine surgery history was defined by the presence of at least one diagnostic (International Classification of Diseases, 10th Edition; ICD-10) or procedural (Current Procedural Terminology; CPT) code in the patient's medical record indicating a surgery was received prior to CSM (Appendix A). In the US, CSM delivery was identified by procedure codes 98940, 98941, and 98942, specific to chiropractic spinal manipulation [15]. Radiofrequency ablation, cryoablation, thermal ablation, and sacroiliac joint surgeries were not considered spine surgeries as part of this study; however, patients undergoing these procedures were not necessarily excluded if they also received spine surgery.

The size and makeup of the cohort with previous surgery were explored and compared to the total included CSM cohort concerning demographics, body mass index, and treatments received over a one-year follow-up period after receipt of CSM. We also compared the prevalence of neck and low back pain between cohorts by assessing occurrences of ICD-10 codes M54.2 and M54.5, respectively. According to corresponding ICD-10 and CPT codes, subcategorization of surgery types was performed whenever possible. The frequency of spinal fusion or arthrodesis was identified by any codes M43.2, M96.0, or Z98.1. Treatments reported in the literature for patients with prior spine surgery and ongoing or recurrent pain were used as a guide while analyzing other therapies received during follow-up [2,13,14]. Medications and procedures were identified using Veterans Health Administration National Drug File (VANDF) and CPT codes (Appendix B).

Statistical analysis was conducted in real-time using the TriNetX database platform viewing software which automatically provides means, standard deviations, and frequencies. Between-cohort differences were compared using a χ2 test for categorical variables or independent-sample t-tests for continuous variables (p-values ≤ 0.05 were deemed statistically significant). We did not use propensity matching or another method of confounder adjustment to control for age, sex, or other differences between cohorts. We intended to assess the real-world characteristics of included patients rather than comparing treatment-related outcomes. Therefore, confounder adjustment would skew our results by making the cohorts more similar.

We conducted a sensitivity analysis to determine the relationship between each year and the proportion of CSM patients with a history of spine surgery. This analysis used the same inclusion codes as the main queries; however, each year was examined individually. The search date of the sensitivity analysis was March 28, 2023. Although the 2023 year was not complete, we included data for this year for descriptive purposes.

## Results

Across 14 academic healthcare centers, we identified 81,291 patients who received CSM, of which 8,808 (10.8%) had previous spine surgery. Demographic, anthropometric, and symptom prevalence data for each cohort observed are listed in Table 1. Patients receiving CSM with previous spine surgery differed in several characteristics from the broader CSM population. Those with previous spine surgery were significantly older, female, were more often non-Hispanic/Latino and White, less often Black, and had a greater body mass index (p<0.0001 for each; Table 1). Neck and low back pain were each more prevalent in the cohort with previous spine surgery (p<0.0001).

**Table 1:** Characteristics of patients receiving chiropractic spinal manipulation compared to a subset of these patients with previous spine surgery Chiropractic spinal manipulation (CSM), CSM with prior spine surgery (CSM + surgery)

	CSM	CSM + surgery	p-value
Patients	81,291	8,808	NA
Age	47.8 ± 20.1	56.0 ± 17.1	<0.0001
Sex			
Female	47,750 (59%)	5,551 (63%)	<0.0001
Male	33,538 (41%)	3,257 (37%)	<0.0001
Race			
Black or African American	3,188 (4%)	248 (3%)	<0.0001
White	62,489 (77%)	7,209 (82%)	<0.0001
Asian	763 (1%)	46 (1%)	<0.0001
Unknown	14,558 (18%)	1,277 (14%)	0.0926
Ethnicity			
Hispanic/Latino	1,700 (2%)	172 (2%)	0.3868
Not Hispanic/Latino	66,991 (82%)	7,884 (90%)	<0.0001
Unknown	12,600 (15%)	752 (9%)	<0.0001
Body mass index	29.2 ± 6.8	30.0 ± 6.5	<0.0001
Symptom			
Low back pain	46,381 (57%)	7,504 (85%)	<0.0001
Neck pain	36,212 (45%)	5,436 (62%)	<0.0001

Surgical fusion was reported in 2,599 (30%) patients with previous spine surgery. Other surgeries were difficult to identify individuals, as laminectomy and discectomy procedures overlapped in many cases. Specific diagnosis codes indicate a history of spine surgery without specifying the type of surgery. There were, at most, ten patients (0.1%) with a recorded presence of a spinal cord stimulator (TriNetX rounds single-digit values to ten to protect patient identity).

**Figure 1:**
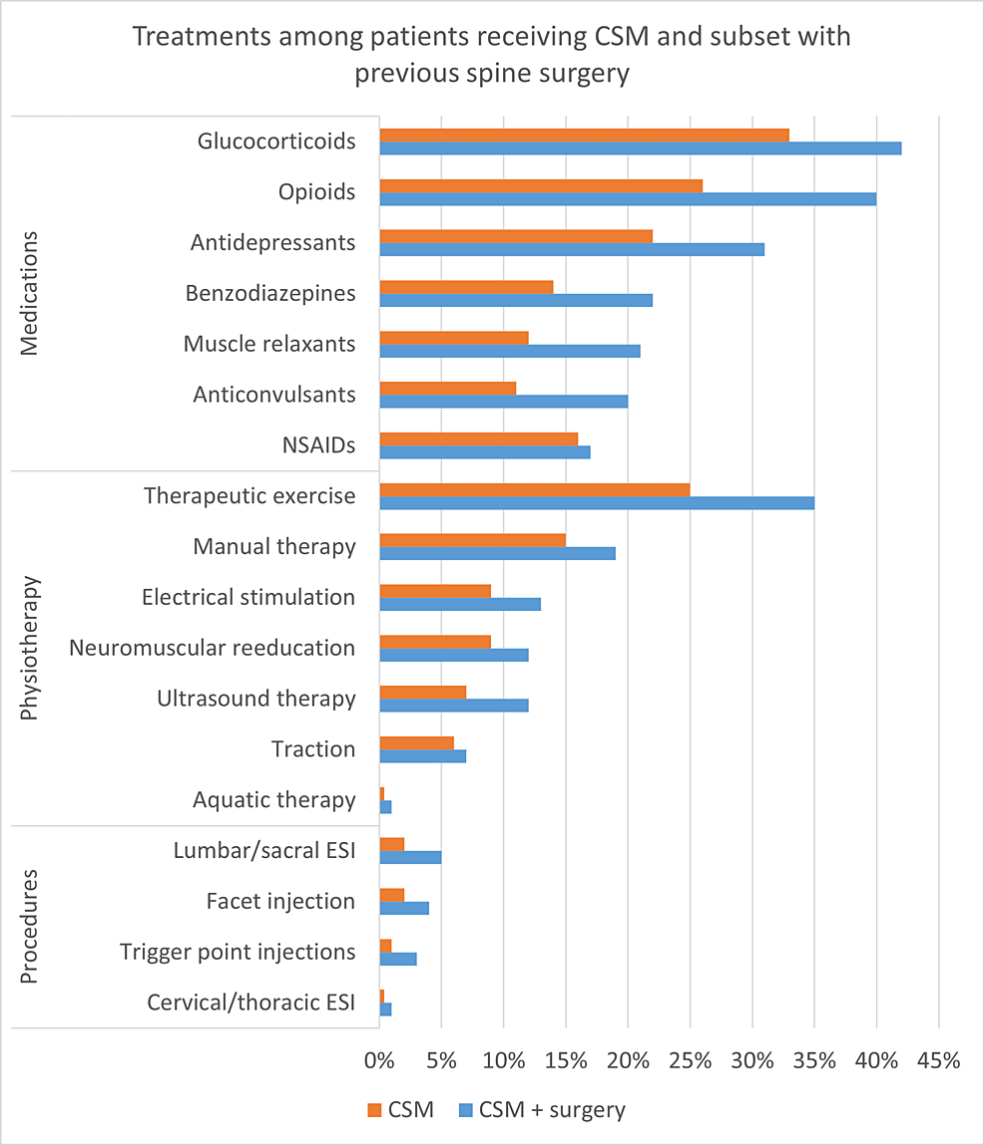
Treatment frequency among patients receiving chiropractic spinal manipulation compared to a subset of these patients with prior spine surgery CSM: Chiropractic spinal manipulation, CSM + surgery: CSM patients with prior spine surgery, NSAIDs: nonsteroidal anti-inflammatory drugs, ESI: epidural steroid injection

Patients in the cohort with prior surgery had a significantly higher frequency of several nonsurgical treatments than the broader CSM cohort over a one-year follow-up from the index date of receiving CSM (Figure 1). First, the cohort with prior surgery had a higher frequency of several physiotherapy treatments, including therapeutic exercise (35% vs. 25%), manual therapy (19% vs. 15%), ultrasound therapy (12% vs. 7%), neuromuscular re-education (12% vs. 9%), electrical stimulation (13% vs. 9%), spinal traction (7% vs. 6%), and aquatic therapy (0.7% vs. 0.4%) (p<0.0001 for each). Second, differences were observed in the prescription rates of several medications, including glucocorticoids (42% vs. 33%), opioids (40% vs. 26%), benzodiazepines (22% vs. 14%), antidepressants (31% vs. 22%), skeletal muscle relaxants (21% vs. 12%), and anticonvulsants (20% vs. 11%) (p<0.0001 for each). There was no difference in nonsteroidal anti-inflammatory medications (17% vs. 16%, p=0.2593). Third, regarding medical procedures, the cohort with prior surgery had a higher frequency of lumbar/sacral epidural steroid injection (5% vs. 2%), facet injection (4% vs. 2%), trigger point injection (3% vs. 1%), and cervical/thoracic epidural steroid injection (0.8% vs. 0.4%) (p<0.0001 for each).

**Figure 2:**
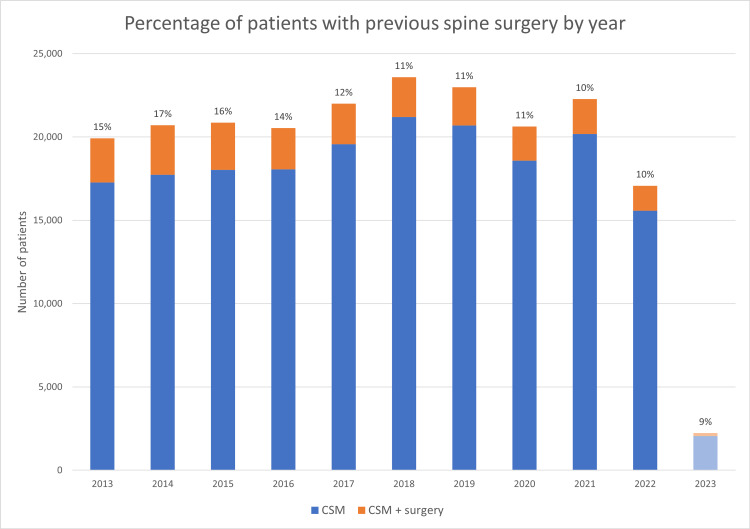
Proportion of patients receiving chiropractic spinal manipulation (CSM) with a history of spine surgery according to year As indicated by the percentage data labels, the proportion of patients receiving CSM with previous spine surgery gradually decreased from its highest value in 2014 to its lowest in 2022-2023. Data from 2023 are stifled as our analysis only included the year's first quarter. CSM: Chiropractic spinal manipulation, CSM + surgery: CSM patients with prior spine surgery

Our sensitivity analysis revealed a decrease in the proportion of patients receiving CSM with a history of spine surgery over time (Figure 2). Of the analyzed years (2013-2023), the year with the greatest proportion of such patients was 2014, wherein 17% of patients receiving CSM had a history of spine surgery. The last year of complete data was 2022, wherein 10% of patients had previous spine surgery.

## Discussion

The current study presents the largest exploration of patients with known spinal surgery receiving CSM. We found that about 11% of patients receiving CSM in large, integrated, academic health centers had at least one previous spine surgery. Compared to all patients receiving CSM, the subset with prior spine surgery was older and had a greater percentage of females and White individuals, among other differences. Patients with prior spine surgery were also more likely to receive several pharmacological and non-pharmacological treatments over one year after receiving CSM.

We identified a high proportion of patients receiving CSM with a history of spine surgery compared to previous similar studies (11% vs. <1%) [2,9,10]. This discrepancy is best explained by a selection bias toward the academic health settings included in our dataset. Only a small percentage (5% or less) of US chiropractors are employed by clinics affiliated with an academic healthcare organization [16]. One study reported that chiropractors within such settings were most often affiliated with physical medicine and rehabilitation or physical therapy departments (40%) [17]. Some chiropractors were alternatively affiliated with orthopedics/sports medicine (11%) or surgical services (6%) [17]. In addition, another study highlighted that chiropractors in integrated settings frequently receive referrals from medical specialists who commonly manage patients with previous spine surgery [18]. This prior study found that 9% of referrals to a chiropractor were from pain management providers, 6% were from physiatrists, and 4% were from orthopedic surgery and neurology each [18]. Therefore, we suspect that departmental affiliations, professional relationships, and specialist referrals within integrated academic healthcare settings may account for the comparatively high number of chiropractic patients with previous spine surgery observed in the present study.

Our sensitivity analysis suggests that the proportion of patients receiving CSM with a history of spine surgery in US academic health centers has decreased over the past decade. However, this finding should be interpreted with caution, considering we cannot determine the reason(s) for this decrease given the scope of our study. Notably, the CSM + surgery population remained similar, while the overall population grew from 2014 to 2018. Further research is needed to elucidate the time trends in treatments for individuals following spine surgery.

The present study better characterizes patients with previous spine surgery receiving CSM. These patients were older (mean age of 56) and often had low back pain. The high proportion of patients with spinal fusion (30%) contrasted two previous case series. Each reported that only 6% of CSM patients had a prior spinal fusion [2,12]. Patients with fusion may have a high prevalence of chronic spinal pain and thus seek care from several provider types, including chiropractors. Spinal fusion surgeries are also typically performed in older patients, which corresponds to the mean age in our CSM + surgery group. Our findings suggest that a prospective study examining CSM efficacy or safety among individuals with previous spine surgery would be most feasible if it targeted a population of older adults with low back pain and prior arthrodesis/fusion.

This study also helps define the overall characteristics of patients receiving CSM, particularly in academic health centers. A previous study suggested that patients visiting a chiropractor preferred to avoid prescription medications [19]. However, in the current study and others examining patients receiving CSM in academic health settings, the frequency of medication use was relatively high [20,21]. Therefore, we suspect that patients receiving CSM in the academic health centers in our current study differ from most patients treated with CSM outside of these clinical settings.

Another pertinent finding from this study is the treatment pathways of patients receiving CSM with prior spine surgery. While many patients received physiotherapy and other conservative interventions, pain medications such as opioids and spinal injection procedures were also common. These treatments were consistent with those used by patients with persistent or recurrent pain after spine surgery previously described in the literature [2,13,14]. While chiropractors may administer certain physiotherapy treatments/modalities, they do not prescribe medications or perform injections, given their scope of practice. These findings suggest that in academic health centers, patients may not solely see a chiropractic provider but are managed as part of a multi-disciplinary care team, including medical physicians and physical therapists.

Given the limited research on the safety and efficacy of CSM for patients with previous spine surgery, further investigation is imperative. Currently, most data regarding the safety of CSM for patients with prior spine surgery have been derived from a limited number of patients in observational case series and reports [1,8]. While adverse events have been reported among patients with previous spine surgery receiving CSM, such as exacerbating symptoms after treatment [1,8], further research is needed to characterize the incidence and severity of such events. In addition, there are no formal guidelines for providing CSM to such patients, including if and when CSM is appropriate after surgery or how it should be applied (i.e., force parameters, preferred method/technique, contraindications) [4]. The present study shows that prior spine surgery among CSM recipients is common in US academic health centers. Accordingly, a large-scale observational or prospective study, potentially using an integrated clinic system or practice-based research network, should be conducted to explore the safety and efficacy of CSM for these patients.

Limitations

Detailed patient-level data were unavailable, given that the information in the dataset was aggregated and de-identified. A chart review design providing such data would have enabled us to more precisely examine the region of the spine where CSM was applied, the technique of CSM (e.g., high-velocity, low-amplitude vs. mobilization), patient symptoms, and surgical details regarding specific spinal level(s) and procedural techniques applied. We could not readily distinguish between the proportion of patients with localized spinal pain (i.e., low back pain, neck pain) and those with radicular pain (e.g., radiculopathy, sciatica). Patients could also have a code specifying segmental and somatic dysfunction instead of low back or neck pain. As the linkage of diagnosis codes could not be determined from the dataset, it was impossible to know the exact indication for CSM per patient (e.g., neck or low back pain). As several spine surgery codes are broadly used for any spinal region, it was impossible to precisely define the relative proportion of patients with cervical, thoracic, lumbar, or lumbosacral surgeries. Patients may have had physiotherapy, medications, or injections for other conditions rather than symptoms related to their spinal complaints. We did not query for procedures specifying the implantation or removal of a neurostimulator. These can be implanted on a trial basis and then removed and thus may not influence CSM care delivery. We omitted dry needling and acupuncture from our analysis, given that there were too few results to be statistically accurate. TriNetX automatically rounded results for these items up to ten - a strategy to de-identify cohorts of less than ten in the analysis. Acupuncture may have been challenging to identify, given the lack of insurance coverage during the data range. The procedure code for dry needling is newer, and delivery of this intervention may have been recorded as a manual therapy procedure. We could not examine several variables related to social determinants of health due to their limited representation in the TriNetX dataset. Some patients had "unknown" race or ethnicity (9% to 18% per cohort), which limits our confidence in between-cohort comparisons of these variables.

## Conclusions

This observational study found that patients with previous spine surgery make up nearly 11% of all patients receiving CSM in large, integrated, academic healthcare institutions in the US. The subset of patients receiving CSM with prior surgery differ characteristically and more frequently use several physiotherapy treatments, medications, and injection procedures compared to the broader CSM population. Our findings could be more broadly generalizable and are likely explained by the clinical settings represented by our dataset. Given the limited research on this topic, further research is needed to examine the safety and efficacy of CSM for patients with prior spine surgery.

## Appendices

Appendix A

**Table 2:** Inclusion codes Current Procedural Terminology (CPT), International Classification of Diseases, 10th Edition (ICD-10); ICD-10 Procedure Coding System (ICD-10-PCS), Systematized Nomenclature of Medicine - Clinical Terms (SNOMED-CT)

Code	Definition	Type
Chiropractic spinal manipulation		
98940, 98941, 98942	Chiropractic spinal manipulation	CPT
Spine surgery		
M43.2	Fusion of spine	ICD-10
M96.0	Pseudarthrosis after fusion or arthrodesis	ICD-10
M96.1	Postlaminectomy syndrome	ICD-10
M96.3	Postlaminectomy kyphosis	ICD-10
M96.4	Postsurgical lordosis	ICD-10
Z96.82	Presence of a neurostimulator	ICD-10
Z98.1	Arthrodesis status	ICD-10
0RB0*, 0RB1*, 0RB3*, 0RB4*, 0RB5*, 0RB6*, 0RB9*, 0RBA*, 0RBB*	Discectomy: occipital-cervical, cervical, cervicothoracic, thoracic, and thoracolumbar	ICD-10-PCS
0SB0*, 0SB2*, 0SB3*, 0SB4*	Discectomy: lumbar and lumbosacral	ICD-10-PCS
0RG0*, 0RG1*, 0RG2*, 0RG4*, 0RG6*, 0RG7*, 0RG8*, ORGA	Spinal fusion: occipital-cervical, cervical, cervicothoracic, thoracic, and thoracolumbar	ICD-10-PCS
0SG0*, 0SG1*, 0SG3*	Spinal fusion: lumbar and lumbosacral	ICD-10-PCS
0RW0*, 0RW1*, 0RW3*, 0RW4*, 0RW5*, 0RW6*, 0RW9*, 0RWA*, 0RWB*	Revision surgery: occipital-cervical, cervical, cervicothoracic, thoracic, and thoracolumbar	ICD-10-PCS
0SW0*, 0SW2*, 0SW3*, 0SW4	Revision surgery: lumbar and lumbosacral	ICD-10-PCS
00NW*, 00NX*, 00NY*	Spinal cord surgery (includes laminectomy): cervical, thoracic, and lumbar	ICD-10-PCS
161619009	History of spinal surgery	SNOMED-CT

 Appendix B

**Table 3:** Treatment codes Current Procedural Terminology (CPT), International Classification of Diseases, 10th Edition (ICD-10); ICD-10 Procedure Coding System (ICD-10-PCS), Systematized Nomenclature of Medicine - Clinical Terms (SNOMED-CT), Veterans Health Administration National Drug File (VANDF)

Code	Definition	Type
Medications		
CN104	Nonsteroidal anti-inflammatory analgesics	VANDF
CN101	Opioid analgesics	VANDF
CN302	Sedatives/hypnotics (e.g., benzodiazepines)	VANDF
CN400	Anticonvulsants (e.g., gabapentin)	VANDF
CN600	Antidepressants	VANDF
HS051	Glucocorticoids	VANDF
MS200	Skeletal muscle relaxants	VANDF
Procedures		
20552, 20553	Trigger point injection	CPT
02583ZZ	Radiofrequency ablation	ICD-10-PCS
62321, 64479, 64480	Cervical/thoracic epidural steroid injection	CPT
64490, 64491, 64493, 64494	Facet injection	CPT
62323, 64483, 64484	Lumbar/sacral epidural steroid injection	CPT
97110	Therapeutic exercise	CPT
97112	Neuromuscular re-education	CPT
97140	Manual therapy	CPT
97113	Aquatic therapy	CPT
97012	Mechanical traction	CPT
97014, 97032	Electrical stimulation	CPT
97035	Ultrasound therapy	CPT
20560, 20561	Dry needling	CPT
97814	Acupuncture	CPT
